# Retrospective cohort study demonstrates that modified CT Severity Index directly correlates with lipase values at or above 600

**DOI:** 10.1016/j.amsu.2020.06.023

**Published:** 2020-06-20

**Authors:** John F. Hamer

**Affiliations:** Department of Radiology, La Grange, Il, 60525, USA; Suburban Radiology, AMITA Health Adventist Medical Center, LaGrange, Il, USA

**Keywords:** Pancreatitis, Lipase, Radiology

## Abstract

**Background:**

Elevated lipase is considered an important biomarker for pancreatitis. The aim of this study was to assess a potential correlation between elevated lipase and characteristic imaging findings, as per the well-established Modified CT Severity index (MCTSI).

**Materials and methods:**

This retrospective, single centre cohort study reviewed the radiologic findings and medical records of 200 consecutive patients with elevated lipase values. Subgroups were then created categorizing patients into low lipase elevation, medium lipase elevation, and high lipase elevation groups. CT exams evaluated by a single fellowship trained radiologist was used to establish MCTSI criteria.

**Results:**

Statistical significance was calculated by an independent statistician using a linear regression model which demonstrated a statistically significant correlation between the high lipase group, (≥600 U/L) and MCTSI with p-value of <0.05. No significant correlation existed between MCTSI and lipase values below 600 U/L.

**Conclusions:**

The previously held notion that lipase values do not correlate with severity of pancreatitis does not hold true for lipase values ≥ 600 U/L. This suggests a likelihood of higher morbidity and may justify earlier use of contrast enhanced CT imaging in patients with pancreatitis whose lipase values are >600 U/L. This needs to be further validated with a multisite blinded prospective study.

## Highlights

• Lipase values and the Modified Computed Tomography Severity Index can both be helpful in determine the severity of pancreatitis.
• Lipase values positively correlate with the severity of pancreatitis for lipase values ≥ 600 U/L.

## Abbreviations

CT: Computed tomography
MCTSI: Modified CT Severity index
ULN: Upper limit of normal
U/L: Units per Litre

## 1 Introduction

Elevated lipase is a common abnormality seen in patients with abdominal pain. This finding is frequently accompanied by computed tomography (CT) imaging. Lipase can be elevated due to pancreatitis or numerous other disease states including other pancreatic, biliary, and gastrointestinal disorders. Previous literature has stated that there is no link between lipase levels and the severity of pancreatitis [[1], [2], [3]].

The Modified CT Severity index (MCTSI) uses an imaging scoring system that has been established to correlate with pancreatitis severity and overall mortality and has been shown to predict the need for interventional procedures [4]. A correlation between lipase values and MCTSI would provide indirect evidence of a correlation between lipase values and the severity of pancreatitis.

The American College of Gastroenterology (ACG) guidelines for the diagnosis of acute pancreatitis includes two of three of the following: 1) an elevated serum lipase or amylase level at least three times the normal value, 2) characteristic radiologic imaging findings, and 3) typical abdominal pain [5]. This study evaluated for a potential correlation between elevated lipase and characteristic imaging findings as per the established MCTSI [6].

We hypothesised that there is a positive correlation between lipase level and the severity of pancreatitis as measured on CT. This retrospective study evaluated for a correlation between lipase values and the severity of pancreatitis as measured by the MCTSI.

## 2 Methods

### 2.1 Ethical approval

The research protocol, developed by the primary investigator, was approved by the medical centre's institutional review board under the expedited review category of research activities that present no more than minimal risks to patients, detailed in 45 CFR 46.110. This study has been registered under the UIN researchregistry5651. This work has been reported in line with the STROCSS criteria [7].

### 2.2 Patients

This retrospective correlative study investigated the medical records and reviewed the radiology findings of 200 consecutive patients with elevated lipase levels at a 196-bed community hospital in the suburbs of Chicago. Lipase values were measured at a single on-site laboratory using Cobas 6000 analyzer series (Roche Diagnostics Ltd., Basel, Switzerland). Patient lipase data that had been measured between January 1, 2015 and December 31, 2017 were collected. All patients with lipase values less than 200 U/L were excluded from study participation. Lipase levels were reported up to 600 U/L; if the lipase level was greater than 600 U/L, it was recorded as > 600 U/L. For patients who had more than one lipase value, only the highest lipase value that had occurred within 24 h of CT imaging was included for analysis. If no CT had been performed, then the highest lipase value was recorded; however, the data was excluded from analysis for the study's primary focus of comparing lipase to MCTSI scores, but the data was analysed for secondary areas of interest, such as mortality rate. Any data from hospitalization of the same patient with lower lipase values were disregarded.

The lipase values were classified into low lipase elevation group (200–400 U/L), moderate elevation group (400–600 U/L), and high elevation group (≥600 U/L). Lipase values were entered into Excel (Microsoft, Seattle, WA) along with patient variables including age, sex, total MCTSI, degree of pancreatic inflammation, pancreatic necrosis, extrapancreatic complications, pseudoaneurysm formation, frequency of patient transfer, and in-patient mortality rate.

### 2.3 Outcome variables

The primary outcome variable was the MCTSI score. Secondary outcome measures included aetiology of elevated lipase, frequency of patient transfer, in-patient mortality rate, and the rate of radiologist agreement. MCTSI were based on previously established criteria with a score of 0–4 for pancreatic inflammation, 0–4 for pancreatic necrosis, and 0–2 for extrapancreatic complications [6].

Ancillary areas of interest were identifying aetiologies of elevated lipase in a community hospital setting with review of the in-patient mortality rate.

All data was obtained from Centricity EMR (GE Healthcare, Chicago, IL) and entered into Excel using a unique medical record number (MRN) to ensure that no patient was included more than once during subsequent admissions. For each patient, radiology studies from that admission were reviewed by a single fellowship trained radiologist with more than 15 years of experience. Patients whose contrast enhanced CT had been completed within 24 h of lipase collection were given an MCTSI score by the interpreting radiologist. If no contrast had been given or contrast had been given more than 24 h after the abnormal lipase detection, all data except the MCTSI were entered. All enhanced CT Images were reviewed on picture archiving and communication system workstation (IMPAX, Agfa Healthcare, Belgium). Discrepancies between the original interpreting radiologist and reviewing radiologist were discussed with the initial interpreting radiologist. Final interpretation was made by consensus. The rate of radiologist agreement of MCTSI criteria was evaluated.

Patient diagnosis of pancreatitis or other diseases were based on medical records and discharge reports from the time of hospitalization and were also entered into the spreadsheet. All patient data was obtained regardless of pancreatitis diagnosis, along with the reported lipase elevated aetiology when it was recorded in the medical record.

All CTs were performed on a multidetector scanner using either GE 64 slice CT scanner (GE Healthcare, Chicago, IL) or a Toshiba Aquilion 132 slice scanner (Toshiba, Tokyo, Japan). Contrast enhanced images were obtained after a delay of 80 s post administration of 100 mL of Omnipaque 350 injected at a rate of 4 mL/s.

### 2.4 Statistical analysis

For comparison purposed three lipase subgroups were created including high (>600 U/L), moderate (400–600 U/L), and lower elevated (200–400 U/L) lipase groups. Statistical significance was calculated by an independent statistician from Loyola University Chicago using R Core Team (2012, R Foundation for Statistical Computing, Vienna, Austria.) software and multiple linear regression with independent variables including variables for high (>600 U/L), moderate (400–600 U/L), and lower elevated lipase (200–400 U/L) using a p-value of 0.05.

## 3 Results

This study included 108 men and 92 women with an average age of 63.0 years (range, 13–98 years). The high lipase subgroup (≥600 U/L) comprised 49% or 98 patients. The medium lipase subgroup (400–600 U/L) comprised 18.5% or 37 patients. The lowest lipase group (200–400 U/L) comprised 32.5% or 65 patients.

**Fig. 1:**
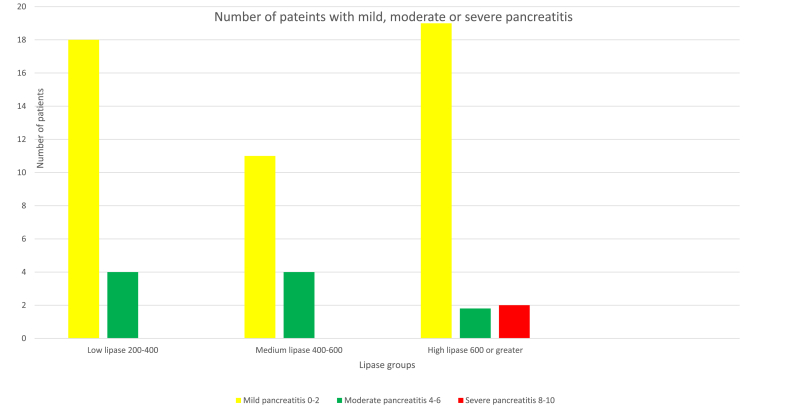
Number of patients with mild, moderate or severe pancreatitis based on MCTSI scores and subdivided into low, medium, or high lipase lab values.

**Fig. 2:**
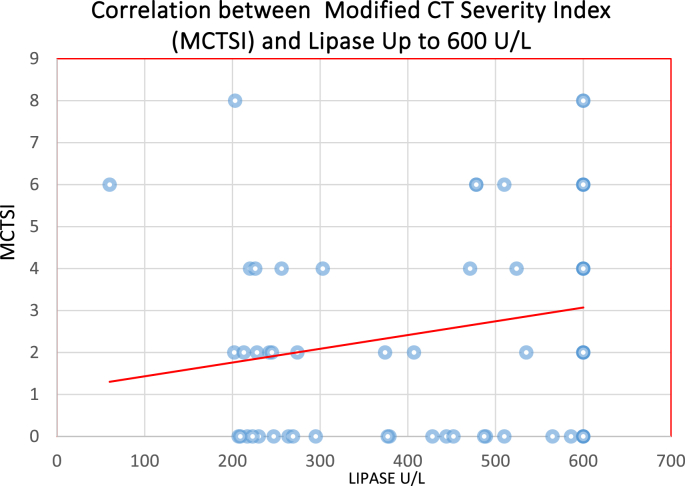
Linear regression demonstrating a positive correlation between lipase and Modified CT Severity Index.

There were 51 contrast enhanced CTs performed in the high lipase group with an average MCTSI of 3.17, 37 contrast enhanced CTs in the medium lipase group with an average MCTSI of 1.60, and 23 contrast enhanced CTs in the low lipase group with average MCTSI of 1.65. The correlation between the high lipase group and MCTSI was statistically significant (Fig. 1, Fig. 2, p < 0.05). There were no significant correlations between the medium and low subgroups and MCTSI.

Of the high lipase group, 74.5% (73 of 98) patients had been diagnosed during that hospitalization with pancreatitis. Twenty of these were noted to be multifactorial, 19 alcohol related, 17 gallstone related, 9 choledocholithiasis, 6 cholecystitis, 6 hypertriglyceridemia, 5 pancreas divisum, 5 medication related, 4 post procedure related after ERCP, and 3 or less autoimmune, viral, biliary stricture, post traumatic, pancreatic cancer, ampullary cancer, pancreatic insufficiency, GI bleed, or diarrhoea related. Included in the high lipase group were 19 non-pancreatitis related aetiologies: 3 multifactorial, 2 pancreatic cancer, 2 choledocholithiasis without pancreatitis, and 3 uncertain aetiology.

The high lipase groups also had increased secondary outcomes in comparison to the other groups. Hospitalization mortality rate for the high lipase group was 1.05% (one patient); transfer to Hospice 1.05% (one patient); transfer to other medical facility 3.15% (three patients); outpatient medical work up was 2.1% (two patients).

## 4 Discussion

In a departure from most current literature, this data suggested that lipase values > 600 U/L may be helpful in determining the severity of pancreatitis. A paediatric study with similar results stated that lipase levels greater than 7 times the upper limit of normal (ULN) within 24 h of presentation were associated with severe acute pancreatitis [8]. That conclusion parallels that of this study likely due to categorizing lipase levels into subgroups, thus showing a significant difference only in the highest lipase group.

This result will be helpful to clinicians treating pancreatitis patients with a lipase value of >600 U/L by aiding with patient risk stratification. Specifically, it will help justifying imaging earlier in the course of disease with intravenous contrast enhanced CT in patients with lipase of >600 U/L and alternatively justify delaying early imaging on patients with lipase of <600 U/L. Future investigation should focus on more specific subdividing of elevated lipase values > 600 U/L into groups, such as lipase values of 600–1000 U/L and >1,000 U/L which is possible in many clinical laboratories.

Study limitations include a retrospective study design, a medium sized sample, and not delineating between new acute pancreatitis versus acute on chronic pancreatitis. In addition, the reviewing radiologist was not blinded to patient data. A prospective study addressing these deficiencies may be helpful.

## 5 Conclusion

This study confirms the importance of CT with contrast for patients with elevated lipase levels (≥600 U/L). Elevated lipase (≥600 U/L) was positively correlated with elevated MCTSI and may need earlier imaging compared to patients with lower levels of lipase elevation. This retrospective study questions the previously stated conclusion that lipase levels do not correlate with the severity of pancreatitis and are not predictive of complications. A further prospective study should focus on subcategorization of higher lipase values and the potential correlation with the severity of pancreatitis and imaging findings.

## Ethical approval

AMITA Health IRB 2018-0059-02.

## Sources of funding

Primary investigator paid costs of independent statistician Vincenzo Palazeti from Loyola University Chicago for statistical analysis and Editage (www.editage.com) for manuscript editing services.

## Author contribution

Dr. John Forrest Hamer-study concept/design/data collection/analysis/manuscript writing and editing.

Statistician Vincenzo Palazeti from Loyola University Chicago for statistical analysis.

Editage (www.editage.com) for manuscript editing services.

## Registration of research studies

Research Registry, IJS Publishing Group Ltd.

UIN researchregistry5651.

https://www.researchregistry.com/browse-the-registry#home/?view_2_search=5651&view_2_page=1.

## Guarantor

Dr. John Forrest Hamer MD.

## Provenance and peer review

Not commissioned, externally peer reviewed.

## Financial

Author has no financial or non-financial interest in the subject matter of this unfunded manuscript.

## Consent

IRB approval.

## Declaration of competing interest

There are no conflicts of interest to report.

Costs of study registration, editing, and statistical review were paid by the primary investigator.
